# Correlation and predictive value of oxygenation and oxygen saturation indices in extremely preterm infants: a prospective study

**DOI:** 10.3389/fped.2025.1476885

**Published:** 2025-02-17

**Authors:** Ibrahim Alanazi, Saleh S. Algarni, Saad Alshreedah, Naif Alotaibi, Mohammed Sufyani, Sami S. Alanazi, Abeer H. Alharthi, Abadi Ghazwani, Omar M. Almutairi, Maryam Alkaabi, Abdulaziz Homedi, Ibrahim Ali, Mohammed Khawaji, Saif Alsaif, Kamal Ali

**Affiliations:** ^1^ Neonatal Intensive Care Department, King Abdulaziz Medical City-Riyadh, Ministry of National Guard Health Affairs, Riyadh, Saudi Arabia; ^2^ Department of Respiratory Therapy, College of Applied Medical Sciences, King Saud bin Abdulaziz University for Health Sciences, Riyadh, Saudi Arabia; ^3^ King Abdullah International Medical Research Center, Riyadh, Saudi Arabia

**Keywords:** oxygenation, index, preterm, correlation, prediction, outcome

## Abstract

**Aims:**

This study aims to evaluate the association between the Oxygenation Index (OI) and the Oxygen Saturation Index (OSI) in extremely preterm infants. In addition, the study seeks to determine the predictive value of these indices for mortality in the first 7 days and Bronchopulmonary Dysplasia (BPD) at 36 weeks postmenstrual age (PMA).

**Methods:**

This is a prospective observational study conducted at King Abdulaziz Medical City, Riyadh between October 2023 and May 2024, involving extremely preterm infants with clinical and ventilator data collected during the first 7 days of life. The predictive capabilities of OI and OSI for mortality within the first 7 days and BPD at 36 wks. PMA were assessed using Area Under the Curve (AUC) analysis, while associations between indices were explored through Spearman's correlation coefficient.

**Results:**

The study included 85 infants with a mean birth weight of 856 grams (SD = 243) and a mean gestational age of 26 weeks (SD = 1.8). There was a strong positive correlation between OI and OSI overall (*r* = 0.848, *p* < 0.001, *n* = 85), with similar findings in both surviving (*r* = 0.831, *p* < 0.001, *n* = 71) and non-surviving groups (*r* = 0.896, *p* < 0.001, *n* = 14). Bland-Altman plots showed a mean difference of 3 between OI and OSI for all infants, with limits ranging from −4 to +8. Tighter agreement was observed in survivors with a mean difference of 2 and limit from −4 to +7, while non-survivors showed a larger mean difference of 4.5 and wider limits of agreement from −8 to +17. Receiver Operating Characteristic (ROC) analysis for survival prediction focused on indices measured within the first 24 h, demonstrating high predictive accuracy. Additionally, the mean daily values for OI and OSI between Day 4 and Day 7 were found to be predictive of BPD at 36 wk. PMA.

**Conclusions:**

Measurements of OI and OSI within the first 24 h effectively predict mortality in extremely preterm infants. Additionally, daily mean values of OI and OSI from day 4 to day 7 were predictive of BPD at 36 weeks PMA. Further research is needed to refine these diagnostic thresholds to enhance neonatal care outcomes.

## Introduction

Neonatal Respiratory Distress Syndrome (RDS) is a common and significant cause of respiratory distress in newborns, typically presenting within the first few hours after birth and most frequently immediately following delivery (1). This condition primarily affects preterm infants and its incidence is inversely related to gestational age (1). Despite advances in neonatal care, including the use of antenatal corticosteroids, surfactant therapy, and modern respiratory support techniques, RDS continues to be a leading cause of morbidity and mortality among preterm infants. Complicating this, Bronchopulmonary Dysplasia (BPD), diagnosed at 36 weeks postmenstrual age (PMA) (2), remains one of the most significant long-term morbidities associated with RDS, underscoring the importance of effective management and prevention strategies in this population.

The Oxygenation Index (OI) and Oxygen Saturation Index (OSI) are critical metrics used in neonatal intensive care units (NICUs) to assess respiratory function and guide therapeutic interventions in critically ill neonates. Both indices have been studied for their predictive capabilities regarding outcomes such as mortality and morbidity in newborns requiring respiratory support (3–6). Previous studies have demonstrated the utility of the OI in assessing the need for and response to surfactant therapy in preterm infants (7, 8). On the other hand, the utilization of the OSI has been growing in both pediatric and adult intensive care settings as an indicator for respiratory failure and lung injury (6, 9, 10).

Studies focusing specifically on the prognostic power of these indices concerning extremely preterm infants are scarce. This study addresses this knowledge gap by focusing on the early measurement of OI and OSI in infants born at less than 28 weeks' gestation. The aim of this study is to evaluates the correlation between the OI and the OSI in extremely preterm infants born at a gestational age of less than 28 weeks. Additionally, the study seeks to determine how initial variations in these indices are associated with mortality in the first 7 days of life and BPD at 36 weeks PMA. Understanding the predictive value of OI and OSI during the initial days post-birth could provide insights that enable more timely and targeted therapeutic interventions.

## Methodology

This prospective observational study was conducted at King Abdulaziz Medical City, Riyadh, Kingdom of Saudi Arabia from October 1, 2023, to May 31, 2024 with the aim of examining the correlations between the OI and the OSI, and their predictive abilities for mortality and BPD at 36 weeks (PMA), in preterm infants with a gestational age of less than 28 weeks at birth. The study protocol received ethical approval from the King Abdullah International Medical Research Centre (KAIMRC) with an approval number of (IRB23R/745/10).

Inclusion criteria were preterm infants with a gestational age of less than 28 weeks and availability of clinical data and ventilator parameters for the first 7 days of life. For infants who did not survive to 7 days, the OI and OSI were calculated from birth until the time of mortality. Exclusion criteria included infants with major congenital anomalies and outborn infants.

Oxygenation and oxygen saturation indices data were collected every time a blood gas was obtained during the first 7 days of life. The OI is calculated using the formula: OI = (MAP × FiO_2_ × 100)/PaO_2_. In this formula, MAP represents the mean airway pressure, and FiO_2_ stands for the fraction of inspired oxygen (11). In the OSI calculation, PaO_2_ is substituted with SpO_2_ (saturation of peripheral oxygen), rendering the OSI formula as OSI = (MAP × FiO_2_ × 100)/SpO_2_ (9, 12). Pulse oximetry measurements in this study were obtained using the Masimo SET® pulse oximeter (Masimo Corporation, Irvine, CA, USA) with Masimo RD SET NeoPT sensors, which are specifically designed for use in neonatal and pediatric patients. Arterial blood gas analysis was performed using the GEM Premier 5,000 blood gas testing system (Instrumentation Laboratory, Bedford, MA, USA). Our department's protocol ensures that all arterial blood gas measurements, the blood sampling source, concurrent oxygen saturation, and the location of the pulse oximeter are recorded in the electronic medical records. Blood gases are obtained from postductal umbilical arterial lines, and a range of OI-OSI pair metrics are evaluated. Typically, infants undergo 4–6 arterial blood gas analyses daily, which may vary depending on clinical stability and necessary adjustments in ventilation. The mean OI and OSI were recorded for each infant when available for the first 7 days of life.

To rigorously assess the immediate postnatal respiratory status and the efficacy of initial clinical interventions, this study specifically analyzed the first 24-hour respiratory indices, including the best, highest, and mean values for OI and OSI. The initial 24 h post-birth represent a critical period where neonatal physiological responses are most dynamic and indicative of both the severity of respiratory challenges and the impact of therapeutic measures. The selection of best and highest indices captures moments of optimal and compromised respiratory function, respectively, providing essential insights into the acute responses to clinical interventions. Meanwhile, the mean indices offer a holistic view of the overall respiratory status within this critical timeframe, establishing a baseline for subsequent evaluations and ongoing management.

In our department, the management RDS in preterm infants adheres to the European consensus guidelines (13). Infants were intubated and ventilated for indications including RDS with failure of non-invasive respiratory support, persistent hypercapnia (PaCO₂ > 65 mmHg), significant respiratory acidosis (pH < 7.2), or profound apnea. For those intubated for stabilization, surfactant is administered, and additional doses are provided based on the severity of RDS. Surfactant delivered via the LISA technique when feasible for infants > 26 weeks' gestation. Continuous Positive Airway Pressure (CPAP) or nasal Intermittent Positive Pressure Ventilation (NIPPV) is initiated early in all at-risk babies. Mechanical ventilation (MV) employs lung-protective modes, aiming to minimize ventilation duration, and tolerate moderate hypercarbia while ensuring pH remains above 7.22. Inhaled Nitric Oxide (iNO) is reserved for confirmed pulmonary hypertension. Post-surfactant, babies on MV are transitioned to CPAP or NIPPV. Caffeine is routinely used in infants under 32 weeks, and low-dose dexamethasone is considered for facilitating extubation in infants ventilated for over 1–2 weeks. Oxygen saturation targets are set between 90% and 94%, with alarm limits at 89% and 95% (13). With regard to the definition of BPD, we have used the Jensen et al. criteria for BPD in our study (2). This definition classifies BPD severity in infants at 36 weeks PMA as grade 1 if the infant requires 2 L/min nasal cannula or less, grade 2 if the infant requires more than 2 L/min nasal cannula or other forms of non-invasive ventilation support, and grade 3 if the infant requires invasive mechanical ventilation (2).

Maternal characteristics recorded included history of premature rupture of membranes (PROM) >18 h, hypertensive disorders (chronic hypertension and pregnancy-induced hypertension), diabetes (gestational and pregestational), chorioamnionitis, and use of antenatal steroids. Baseline demographics for each infant included gestational age, birth weight, gender, Apgar scores at 1 and 5 min, and mode of delivery. In addition, the duration of ventilation to extubation and the occurrence of pulmonary air leak were also documented.

### Statistical analysis

Data analysis utilized SPSS software, version 26. The sample size required to detect significant correlations between the OI and OSI, and to examine their predictive ability for adverse outcomes was calculated using power analysis based on earlier research (14). This analysis suggested that a cohort of 85 infants would achieve an 80% at the 5% significance level.

Descriptive statistics were calculated to summarize maternal characteristics, baseline infant's demographics, and clinical outcomes. These included means, standard deviations (SD), medians, and interquartile ranges (IQR) for continuous variables, and frequencies and percentages for categorical variables. The values for OI and OSI were rounded to the nearest integer for clarity and ease of presentation. Spearman's correlation coefficient was used to determine the correlation of OI and OSI, and linear regression techniques were employed to establish an equation representing the association between OSI and OI. The concordance between the OI and OSI data was analyzed using the Bland-Altman method, which involved plotting the difference between the indices against their average value. The consistency range was defined by the mean difference plus or minus two standard deviations, establishing the bounds of agreement (15). Receiver Operating Characteristic (ROC) curves were generated to evaluate the predictive ability of OI and OSI for BPD and mortality, with the area under the curve (AUC) calculated to determine the accuracy of these indices. *P*-value of <0.05 was considered statistically significant.

## Results

Table 1 shows the baseline characteristics noting several key maternal and neonatal factors. Among these, 36% of mothers had a prolonged rupture of membranes (PROM) exceeding 18 h. Maternal hypertension and diabetes were present in 16% and 14% of cases, respectively, while chorioamnionitis was observed in 7% of the mothers. Significantly, 89% of mothers received antenatal steroids, and the same proportion of infants required delivery room intubation. Infants had an average birth weight of 856 grams (SD = 243) and a mean gestational age of 26 weeks (SD = 1.8). Males accounted for 54% of the cohort, with an identical percentage delivered by caesarean section. Apgar scores at one minute averaged 5, with a range from 1 to 8, improving to an average of 7 at five minutes, with a range from 3 to 9. For respiratory support, 47% of the infants received conventional ventilation, 44% underwent HFOV, and 9% were managed with non-invasive ventilation. The median duration of ventilation was 10 days, with IQR (3, 17) days. Survival rate stood at 84%, and 62% of those surviving infants were diagnosed with BPD at 36 PMA, while air leaks were noted in 9% of the infants.

**Table 1:** Baseline characteristics of study population.

Variable	Value (Mean ± SD or %/*n*), Median (IQR)
PROM (>18 h)	31 (36%)
Maternal hypertension	14 (16%)
Maternal diabetes	12 (14%)
Maternal chorioamnionitis	6 (7%)
Postnatal steroids	76 (89%)
Birth weight (grams)	856 (243)
Gestational age (weeks)	26 (1.8)
Gender (male)	46 (56%)
Mode of delivery (caesarean)	46 (54%)
Apgar score at 1 min	5 [1, 8]
Apgar score at 5 min	7 [3, 9]
Delivery room intubation	76 (89%)
Mode of ventilation: Conventional ventilation	40 (47%)
High Frequency oscillatory ventilation (HFOV)	37 (44%)
Non-invasive ventilation	8 (9%)
Duration of ventilation (days)	10 [3, 17]
Survival	71 (84%)
BPD at 36 wks. PMA	44 (62%)
Pneumothorax	8 (9%)

PROM, prolonged rupture of membranes; BPD, bronchopulmonary dysplasia; PMA, postmenstrual age.

Table 2 shows the Spearman's rho correlations between the OI and OSI across three distinct groups: all infants, survivors, and non-survivors. For the entire cohort of infants (*n* = 85) with a total of 1,343 samples, there was a strong positive correlation between the two indices (Spearman's rho = 0.848, *p* < 0.001). When examining the subset of survivors (*n* = 71), encompassing 1,142 samples, the correlation coefficient remains strong (Spearman's rho = 0.831, *p* < 0.001). This consistent high correlation among survivors indicates that both OI and OSI reliably track similar respiratory function metrics. In the group of non-survivors (*n* = 14), consisting of 201 samples, the correlation between OI and OSI is even stronger (Spearman's rho = 0.896, *p* < 0.001). Overall, the data demonstrate consistently strong and statistically significant correlations between OI and OSI across all groups. These findings suggest that both indices are closely related in their assessment of respiratory function in critically ill neonates, regardless of their survival outcome.

**Table 2:** Correlations of the OI and OSI for all infants, survivors and non-survivors.

Group	Number of OI/OSI pairs	OI/OSI correlation coefficient (r)	Sig. (2-tailed)
All infants (*n* = 85)	1,343	0.848^a^	<0.001
Survivors (*n* = 71)	1,142	0.831^a^	<0.001
Non-survivors (*n* = 14)	201	0.894^a^	<0.001

OI, oxygenation index; OSI, oxygen saturation index.

^a^ Correlation is significant at the 0.01 level (2-tailed).

Table 3 presents daily OI and OSI values for survivors and non-survivors over the first seven days. On Day 1, survivors had a median OI of 4.0 [3.0, 8.0] compared to 24 [9.0, 47] in non-survivors (*p* < 0.001), and a median OSI of 3.0 [2.0, 4.0] compared to 11 [7.0, 28] in non-survivors (*p* = 0.017). On Day 2, survivors had a median OI of 4.0 [3.0, 6.0] compared to 7.0 [4.0, 14] in non-survivors (*p* = 0.014), and a median OSI of 3.0 [2.0, 4.0] compared to 6.0 [3.0,10] in non-survivors (*p* = 0.009). From Day 3 onward, OI and OSI differences between survivors and non-survivors were not statistically significant. For example, on Day 3, survivors had a median OI of 4.0 [3.0, 7.0] compared to 6.0[4.0, 8.0] in non-survivors (*p* = 0.775), and a median OSI of 3.0 [2.0, 4.0] compared to 4.0 [3.0,7.0] in non-survivors (*p* = 0.358). Similar patterns without significant differences continued through Days 4, 5, 6, and 7. For instance, on Day 7, survivors had a median OI of 4.0 [3.0, 7.0] compared to 9.0 [7.0, 16] in non-survivors (*p* = 0.386), and a median OSI of 2.0 [1.0, 5.0] compared to 3.0 [2.0, 9.0] in non-survivors (*p* = 0.556).

**Table 3:** Comparison of daily oxygenation and oxygen saturation indices between survivors and Non-survivors during the first week.

	Survivors	Non-survivors	*P*-value
Day 1 OI	4.0 [3.0, 8.0]	24 [9.0, 47.0]	<0.001
Day 1 OSI	3.0 [2.0, 4.0]	11 [7.0, 28.0]	<0.017
Day 2 OI	4.0 [3.0, 6.0]	7.0 [4.0, 14.0]	0.014
Day 2 OSI	3.0 [2.0, 4.0]	6.0 [3.0, 10.0]	0.009
Day 3 OI	4.0 [3.0, 7.0]	6.0 [4.0, 8.0]	0.775
Day 3 OSI	3.0 [2.0, 4.0]	4.0 [3.0, 7.0]	0.358
Day 4 OI	5.0( [4.0, 7.0]	8.0 [5.0, 15.0]	0.197
Day 4 OSI	3.0 [2.0, 4.0]	5.0 [3.0, 9.0]	0.176
Day 5 OI	6.0 [4.0, 7.0]	8.0 [5.0, 10.0]	0.307
Day 5 OSI	3.0 [2.0, 4.0]	5.0 [3.0, 7.0]	0.358
Day 6 OI	5.0 [4.0, 7.0]	13 [9.0, 15.0]	0.065
Day 6 OSI	3.0 [2.0, 4.0]	9.0 [3.0, 10.0]	0.075
Day 7 OI	4.0 [3.0, 7.0]	9.0 [7.0, 16.0]	0.386
Da 7 OSI	2.0 [1.0, 5.0]	3.0 [2.0, 9.0]	0.556

OI, oxygenation index; OSI, oxygen saturation index.

**Figure 1:**
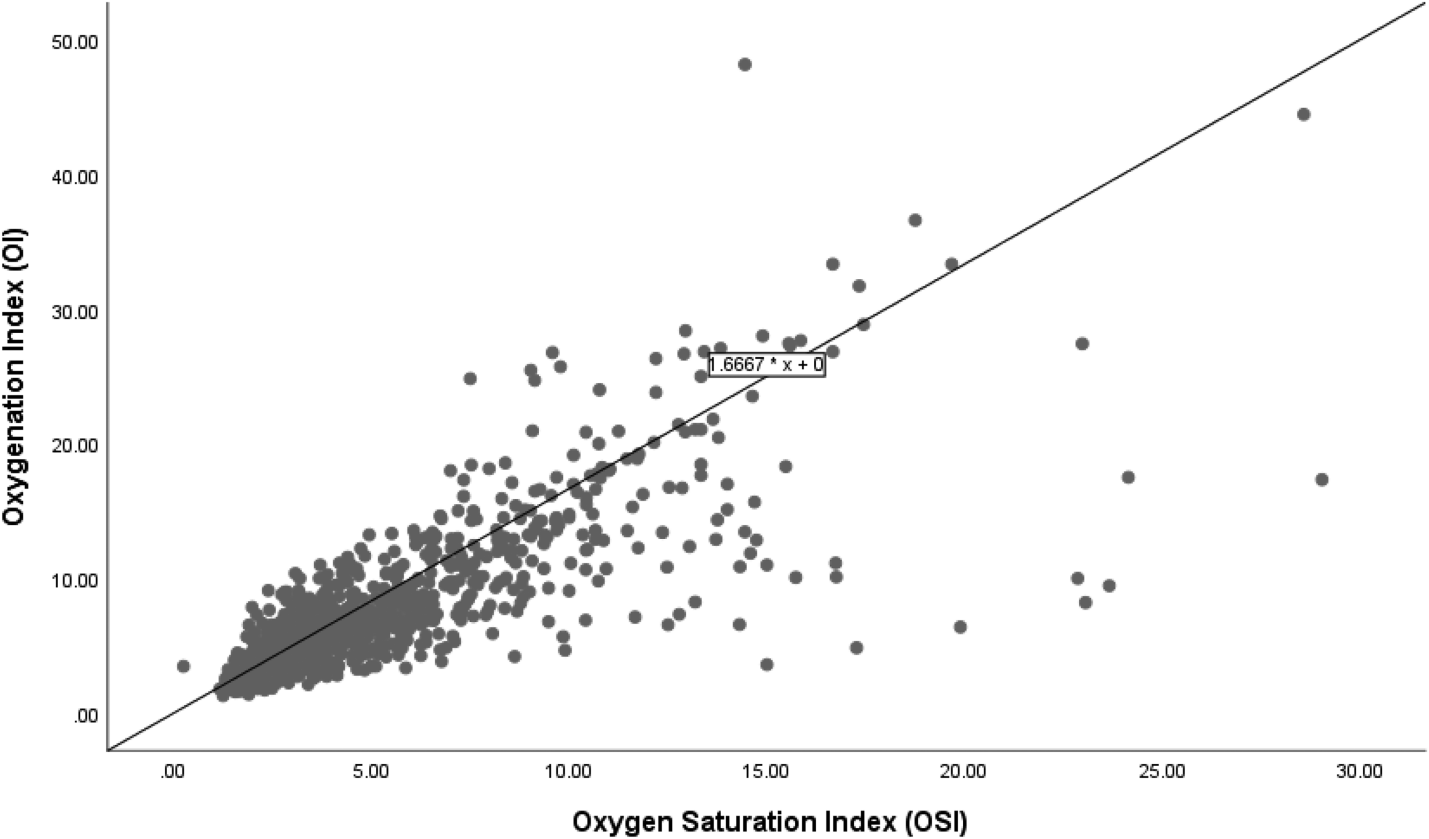
Correlation of oxygenation and oxygen saturation indices in all infants.

Figure 1 shows a scatter plot illustrating the relationship between the OI and OSI in a cohort of 85 infants. The plot demonstrates a positive linear relationship between OI and OSI, with the regression equation OI = 1.667 × OSI, indicating that for every unit increase in OSI, OI increases by 1.667 units, with no baseline offset; meaning that if OSI is zero, OI is also zero.

**Figure 2:**
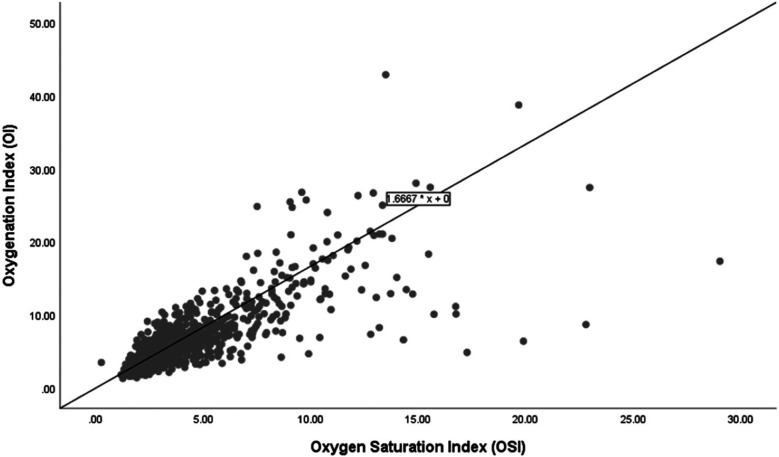
Correlation of oxygenation and oxygen saturation indices among survivors.

Figure 2 shows the scatter plot and linear regression line between the OSI and the OI for the 71 infants who survived. The regression equation OI = 1.6667  ×  OSI delineates this relationship, indicating that for every unit increase in OSI, the OI increases by approximately 1.6667 units. In this group, the regression equation demonstrates a similar increase in OI with rising OSI compared to the overall group. This relationship demonstrates no baseline offset, implying that an OSI of zero corresponds to an OI of zero.

**Figure 3:**
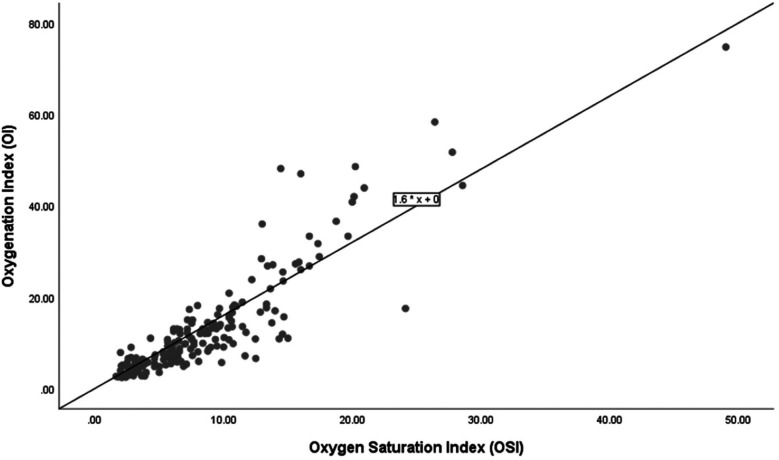
Correlation of oxygenation and oxygen saturation indices among non-survivors.

Figure 3 shows a scatter plot depicting the relationship between OSI and OI among 14 infants who did not survive. The linear regression equation OI = 1.6  ×  OSI is displayed.

**Figure 4:**
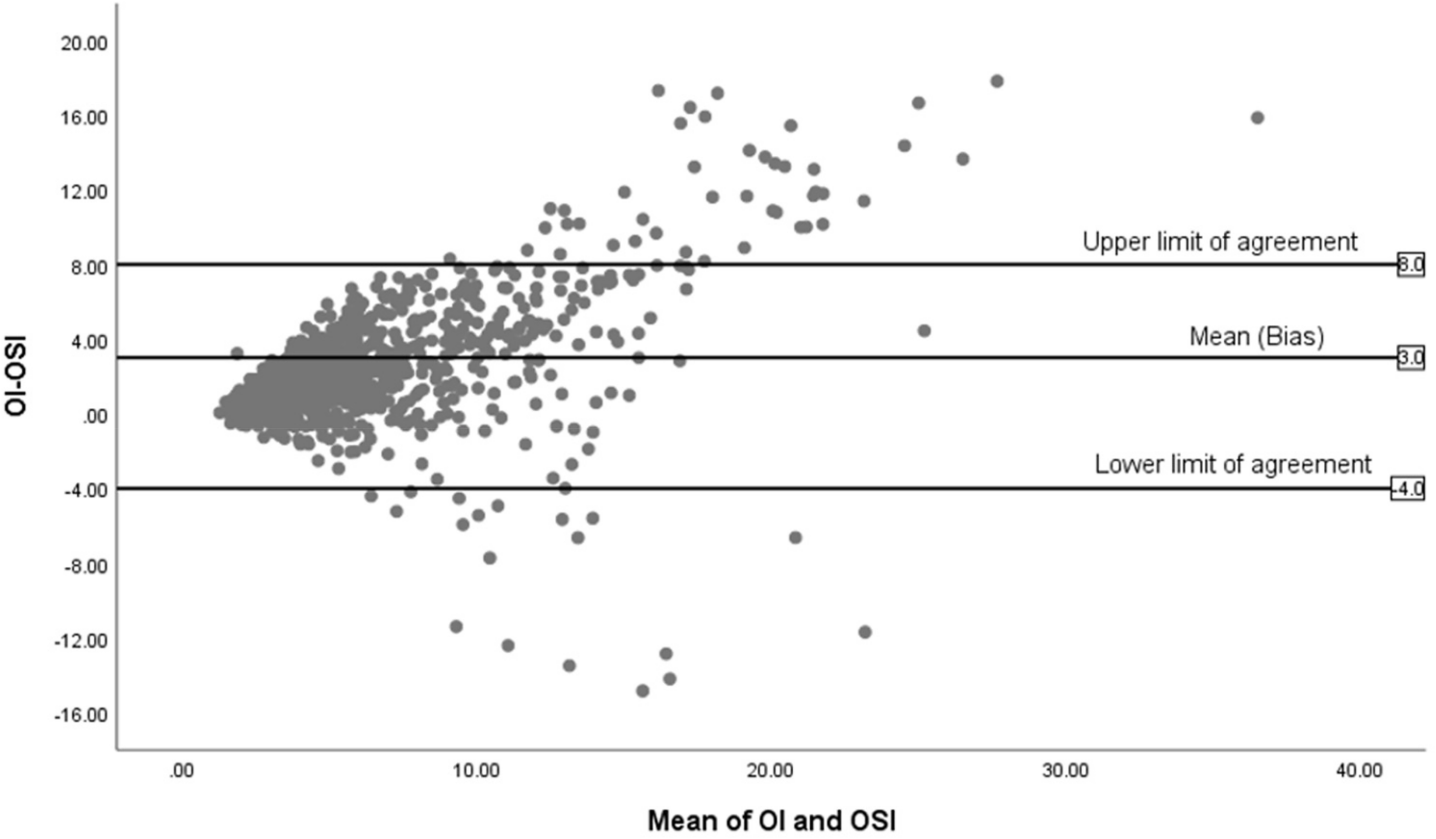
Bland-Altman plot of OI and OSI for all infants (*n* = 85).

Figure 4 displays the Bland-Altman plot for the relationship between the OSI and the OI across the entire cohort in the study (*n* = 85), showing a mean difference of 3. The limits of agreement span from −4 to +8, reflecting moderate variability.

**Figure 5:**
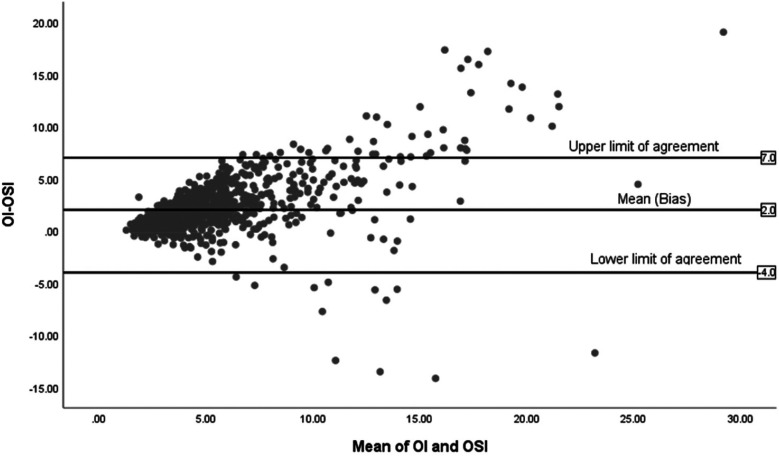
Bland-Altman plot of OI and OSI for survivors (*n* = 71).

Figure 5 illustrates the Bland-Altman plot for 71 surviving infants, showing a mean difference of 2, which indicates a more stable agreement compared to the overall group. The limits of agreement span from −0.4 to +7, suggesting less variability among survivors.

**Figure 6:**
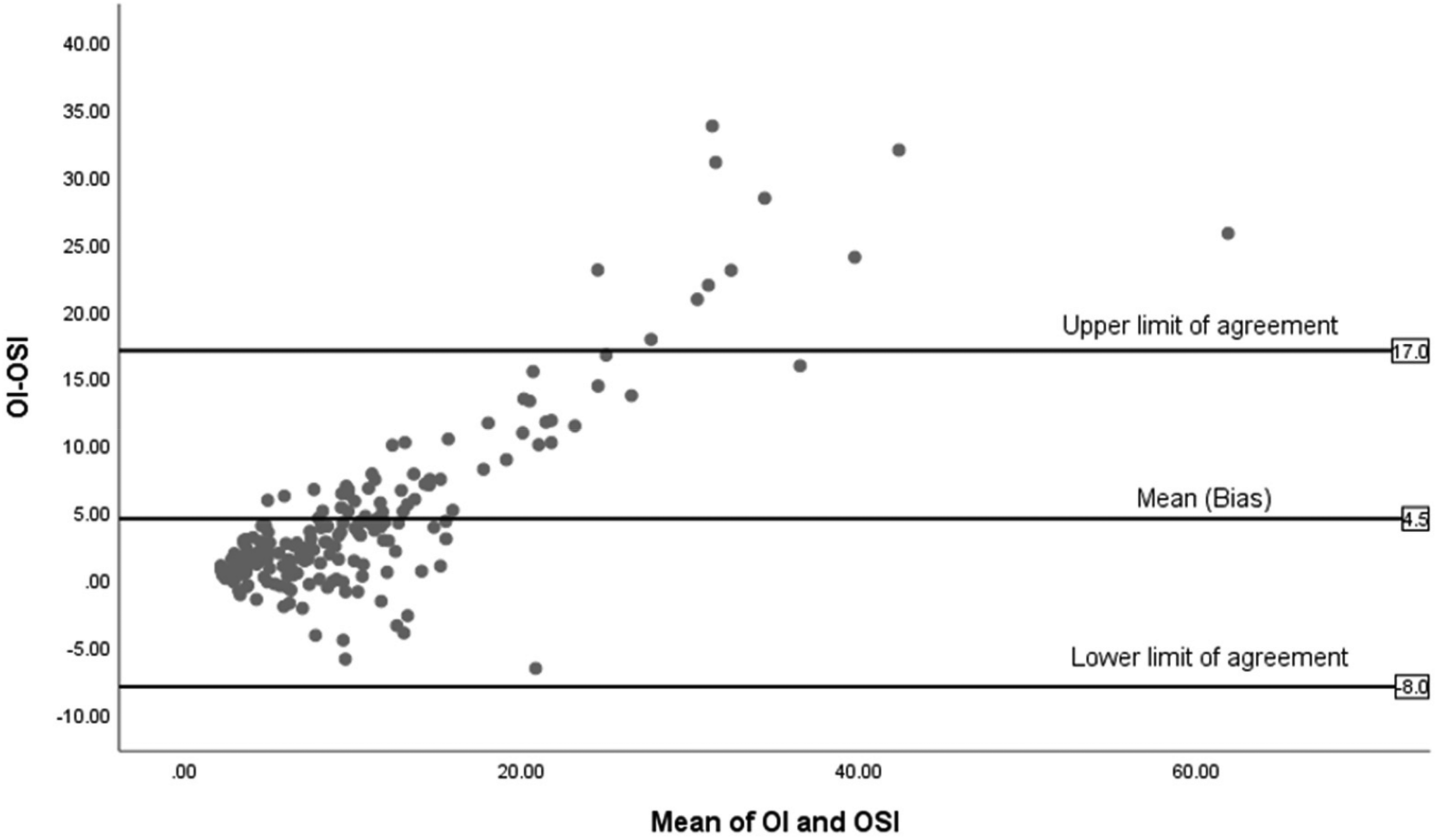
Bland-Altman plot of OI and OSI for non-survivors (*n* = 14).

Figure 6, illustrates the Bland-Altman plot for the 14 non-surviving infants, showing fewer data points with a wide spread, especially towards higher mean values. The mean difference is significantly higher at 4.5 and the limits of agreement are broad, from −8 to +17, reflecting significant variability.

Table 4 presents the Spearman's rho correlation coefficients among various day 1 respiratory indices for the 85 infants in the study. The indices include mean, best (lowest) and highest day 1 OI, in addition to the mean, best (lowest), and highest day 1 OSI. All correlations are significant at the 0.01 level (2-tailed). Mean day 1 OI shows strong positive correlations with best day 1 OI (0.853), highest day 1 OI (0.965), mean day 1 OSI (0.900), best day 1 OSI (0.839), and highest day 1 OSI (0.856). This indicates that as mean day 1 OI increases, the other indices also increase, reflecting consistent relationships among these measures. Best day 1 OI is strongly correlated with highest day 1 OI (0.752), mean day 1 OSI (0.816), best day 1 OSI (0.834), and highest day 1 OSI (0.748). Highest day 1 OI shows robust correlations with mean day 1 OSI (0.875), best day 1 OSI (0.789), and highest day 1 OSI (0.871). Mean day 1 OSI has strong correlations with best day 1 OSI (0.901) and highest day 1 OSI (0.969). Best day 1 OSI is highly correlated with highest day 1 OSI (0.829). The high correlations among these indices suggest they are reliable and interrelated measures of neonatal respiratory status.

**Table 4:** Correlations of the first day mean, best and highest oxygenation and oxygen saturation indices.

			Mean OI day 1	Best OI day 1	Highest OI day 1	Mean OSI day 1	Best OSI day 1	Highest OSI day 1
Spearman's rho	Mean OI day 1	Correlation Coefficient	1.000	0.853^a^	0.965^a^	0.900^a^	0.839^a^	0.856^a^
		Sig. (2-tailed)		<0.001	<0.001	<0.001	<0.001	<0.001
		N	85	85	85	85	85	85
	Best OI day 1	Correlation Coefficient	0.853^a^	1.000	0.752^a^	0.816^a^	0.834^a^	0.748^a^
		Sig. (2-tailed)	<0.001		<0.001	<0.001	<0.001	<0.001
		N	85	85	85	85	85	85
	Highest OI day 1	Correlation Coefficient	0.965^a^	0.752^a^	1.000	0.875^a^	0.789^a^	0.871^a^
		Sig. (2-tailed)	<0.001	<0.001		<0.001	<0.001	<0.001
		N	85	85	85	85	85	85
	Mean OSI day 1	Correlation Coefficient	0.900^a^	0.816^a^	0.875^a^	1.000	0.901^a^	0.969^a^
		Sig. (2-tailed)	<0.001	<0.001	<0.001		<0.001	<0.001
		N	85	85	85	85	85	85
	Best OSI day 1	Correlation Coefficient	0.839^a^	0.834^a^	0.789^a^	0.901^a^	1.000	0.829^a^
		Sig. (2-tailed)	<0.001	<0.001	<0.001	<0.001		<0.001
		N	85	85	85	85	85	85
	Highest OSI day 1	Correlation Coefficient	0.856^a^	0.748^a^	0.871^a^	0.969^a^	0.829^a^	1.000
		Sig. (2-tailed)	<0.001	<0.001	<0.001	<0.001	<0.001	
		N	85	85	85	85	85	85

OI, oxygenation index; OSI, oxygen saturation index.

^a^ Correlation is significant at the 0.01 level (2-tailed).

Table 5 provides an analysis of the Area Under the Curve (AUC) for various respiratory indices from day 1 to predict survival among 85 infants, with 71 survivors and 14 non-survivors. The Mean day 1 OI shows strong predictive ability with an AUC of 0.848 (95% CI: 0.712–0.983, *p* < 0.001), indicating it is a reliable predictor of survival. The Mean day 1 OSI also demonstrates strong predictive power, with an AUC of 0.845 (95% CI: 0.711–0.978, *p* < 0.001). The best (lowest) values for OI and OSI on day 1 further support their predictive utility, with AUCs of 0.829 and 0.860, respectively. The highest values for OI and OSI on day 1, with AUCs of 0.808 and 0.805, respectively, also indicate good predictive capabilities.

**Table 5:** Area under the curve using day 1 oxygenation and oxygen saturation indices for prediction of mortality.

	AUC	*P*-value	95% CI lower bound	95% CI upper bound
Mean OI day 1	0.848 (0.712–0.983)	<0.001	0.712	0.983
Best (lowest) OI day 1	0.829 (0.686–0.971)	<0.001	0.686	0.971
Highest OI day 1	0.808 (0.666–0.950)	<0.001	0.666	0.950
Mean OSI day1	0.845 (0.711–0.978)	<0.001	0.711	0.978
Best (lowest) OI day 1	0.860 (0.750–0.970)	<0.001	0.750	0.970
Highest OSI day1	0.805 (0.649–0.962)	<0.001	0.649	0.962

OI, oxygenation index, OSI, oxygen saturation index; AUC, area under the curve.

Table 6 shows daily OI and OSI values for infants with and without BPD at 36 weeks PMA. On Day 1, the median OI was 5.0 [4.0, 8.0] for the BPD group and 4.0 [3.0, 5.0] for the non-BPD group (*p* = 0.169), while the median OSI was 4.0 [3.0, 5.0] and 3.0 [2.0, 4.0], respectively (*p* = 0.672). From Day 2 onwards, differences in OI and OSI between the two groups were statistically significant. On Day 2, the median OI was 5.5 [3.0, 6.0] vs. 4.0 [3.0, 5.0] (*p* = 0.029) and the median OSI was 4.0 [2.0, 5.0] vs. 2.5 [2.0, 5.0] (*p* = 0.004). On Day 3, the median OI was 6.0 [3.0, 7.0] vs. 4.0 [2.0, 5.0] (*p* = 0.013) and the median OSI was 3.5 [2.0, 5.0] vs. 2.0 [1.5, 3.0] (*p* = 0.001). On Day 4, the median OI was 6.0[4.0, 7.0] vs. 4.0 [2.0, 6.0] (*p* < 0.001) and the median OSI was 3.5 [2.5, 4.0] vs. 2.5 [2.0, 3.5] (*p* < 0.001). On Day 5, the median OI was 7.0 [4.0, 8.0] vs. 4.0 [3.0, 6.0] (*p* = 0.001) and the median OSI was 4.0 [2.0, 5.0] vs. 2.5 [2.0, 3.0] (*p* < 0.001). On Day 6, the median OI was 7.0 [4.0, 8.0] vs. 4.0 [3.0, 5.0] (*p* < 0.001) and the median OSI was 3.0 [2.0, 4.0] vs. 2.0 [1.5, 3.0] (*p* < 0.001). On Day 7, the median OI was 6.0 [3.0, 7.0] vs. 3.0 [2.0, 4.0] (*p* < 0.001) and the median OSI was 3.0 [2.0, 5.0] vs. 2.0 [1.0, 3.0] (*p* < 0.001).

**Table 6:** Comparison of daily oxygenation and oxygen saturation indices between infants with and without BPD at 36 weeks .

	BPD at 36 wks. PMA (*n* = 44)	No BPD (*n* = 27)	*P*-value
OI day 1	5.0 [4.0, 8.0]	4.0 [3.0, 5.0]	0.169
OI day 2	5.5 [3.0, 6.0]	4.0 [3.0, 5.0]	0.029
OI day 3	6.0 [3.0, 7.0]	4.0 [2.0, 5.0]	0.013
OI day 4	6.0 [4.0, 7.0]	4.0 [2.0, 6.0]	<0.001
OI day 5	7.0 [4.0, 8.0]	4.0 [3.0, 6.0]	0.001
OI day 6	7.0 [4.0, 8.0]	4.0 [3.0, 5.0]	<0.001
OI day 7	6.0 [ 3.0–7.0]	3.0 [2.0, 4.0]	<0.001
OSI day 1	4.0 [3.0, 5.0]	3.0 [2.0, 4.0]	0.672
OSI day 2	4.0 [2.0, 5.0]	2.5 [2.0, 5.0]	0.004
OSI day 3	3.5 [2.0, 5.0]	20 [1.5, 3.0]	0.001
OSI day 4	3.5 [2.5, 4.0]	2.5 [2.0, 3.5]	<0.001
OSI day 5	4.0 [2.0, 5.0]	2.5 [2.0, 3.0]	<0.001
OSI day 6	3.0 [2.0–4.0]	2.0 [1.5, 3.0]	<0.001
OSI day 7	3.0 [2.0, 5.0]	2.0 [1.0, 3.0]	<0.001

OI, oxygenation index; OSI, oxygen saturation index; BPD, bronchopulmonary dysplasia; PMA, postmenstrual age.

Table 7 summarizes the area under the curve (AUC) values for predicting BPD at 36 weeks PMA using mean daily OI and OSI from the first week of life. Notably, starting from day 4, both indices show enhanced predictive capabilities. Specifically, the AUC for day 4 OI is 0.708 (95% CI: 0.571–0.845, *p* = 0.014), indicating an increase in its predictive reliability. This trend continues with day 5 OI registering an AUC of 0.685 (95% CI: 0.548–0.822, *p* = 0.028), day 6 OI at 0.749 (95% CI: 0.625–0.873, *p* = 0.003), and day 7 OI at 0.726 (95% CI: 0.599–0.854, *p* = 0.007). For OSI, significant values start from day 4 with an AUC of 0.713 (95% CI: 0.576–0.850, *p* = 0.011), and similar values persist through days 5, 6, and 7, all with *p*-values at or below 0.016. These results highlight the potential of both OI and OSI as reliable indicators for predicting the risk of BPD at 36weeks PMA from the fourth day onwards.

**Table 7:** Area under the curve using daily OI and OSI for prediction of BPD at 36 weeks PMA.

Variable	AUC	*P*-value	95% CI lower bound	95% CI upper bound
Mean day 1 OI	0.638	0.101	0.477	0.800
Mean day 2 OI	0.591	0.279	0.439	0.744
Mean day 3 OI	0.634	0.112	0.480	0.788
Mean day 4 OI	0.708	0.014	0.571	0.845
Mean day 5 OI	0.685	0.028	0.548	0.822
Mean day 6 OI	0.749	0.003	0.625	0.873
Mean day 7 OI	0.726	0.007	0.599	0.854
Mean day 1 OSI	0.628	0.129	0.462	0.794
Mean day 2 OSI	0.613	0.179	0.466	0.761
Mean day 3 OSI	0.661	0.056	0.516	0.806
Mean day 4 OSI	0.713	0.011	0.576	0.850
Mean day 5 OSI	0.703	0.016	0.572	0.834
Mean day 6 OSI	0.703	0.016	0.571	0.835
Mean day 7 OSI	0.707	0.014	0.577	0.838

OI, oxygenation index; OSI, oxygen saturation index; AUC, area under the curve; BPD, bronchopulmonary dysplasia; PMA, postmenstrual age.

## Discussion

Our study of eighty-five extremely preterm infants demonstrates significant correlations between the Oxygenation Index (OI) and the Oxygen Saturation Index (OSI), affirming OSI as a reliable indicator of oxygenation across various clinical conditions. We have shown that the OSI closely mirrors OI in stable infants and provides consistent readings in non-surviving infants experiencing physiological instability. Nevertheless, the Bland-Altman analysis highlights variability in OSI's performance, especially in non-survivors, underscoring the necessity for careful interpretation in neonatal intensive care settings. Additionally, we have also shown that both OI and OSI were predictive of mortality in the first week of life and Bronchopulmonary Dysplasia (BPD) at 36 weeks postmenstrual age (PMA).

The regression analysis depicted in the scatter plots provides a quantitative basis for predicting the OI from the OSI across different subgroups of extremely preterm infants. The relationship between OSI and OI for the entire cohort, survivors, and non-survivors is consistently expressed by the regression equation: OI = 1.66  ×  OSI. This uniformity in the regression coefficient across all groups indicates a strong and consistent predictive power of OSI for OI, suggesting that OSI can reliably indicate oxygenation across a broad range of clinical conditions. The consistent predictive relationship across all subgroups supports the use of OSI as a surrogate measure in clinical settings, reflecting its effectiveness in tracking OI regardless of the patient's survival status. Based on the regression formula (OI = 1.66  ×  OSI), we propose specific OI values for different OSI measurements in extremely preterm infants to aid clinical decision-making. For example, an OSI of 3 predicts an OI of 5, an OSI of 6 corresponds to an OI of 10, and an OSI of 9 leads to an OI of 15. Additionally, an OSI of 12 indicates an OI of 20, an OSI of 15 matches an OI of 25, and an OSI of 24 results in an OI of 40. Nevertheless, we note divergence of OSI and OI in higher values which is primarily due to the nonlinear relationship between SpO_2_ and the PaO_2_ as saturation approaches 100%. At lower OSI levels, changes in SpO_2_ closely mirror changes in PaO_2_, influencing the OI correspondingly. However, at higher OSI levels, minor variations in saturation can cause significant fluctuations in PaO_2_, leading to broader variations in OI.

In a retrospective analysis, Rawat and colleagues (16) examined late preterm and term neonates, identifying a strong correlation between the OSI and OI within a specific OI range. They developed a formula to estimate OI from OSI (OI = 2 × OSI), which differs from the equation (OI = 1.66 × OSI) derived from our study involving extremely preterm infants (16). This variation in regression models may be attributed to differences in gestational and postnatal ages at which OSI and OI were measured. Similarly, Doreswamy et al. conducted a prospective study in neonates, establishing OSI thresholds of 3 and 6.5 to predict OI values of 5 and 15, respectively (17). In another study by Muniraman involving 220 infants, both term and preterm, a robust correlation (*r* = 0.89) between the OI and OSI was observed, which was notably stronger in preterm infants, particularly those under 28 weeks and those between 28 and 33 weeks, with a correlation of 0.93 in both groups (18). Variations between the studies highlight that the developmental stage at assessment significantly impacts the predictive accuracy of such indices, suggesting that adjustments may be necessary when applying these models across diverse neonatal populations.

In the Bland-Altman analysis, the differences in agreement between the OI and OSI across all infants, surviving infants, and non-surviving infants reveal important variations in how these indices perform under different clinical conditions. For all infants, there's a moderate mean difference and a broad scatter of data points, indicating that while OSI generally follows OI, the individual variability is considerable, highlighting the index's general utility but limited precision across a heterogeneous group. For surviving infants, the mean difference is small, suggesting a tighter agreement between OI and OSI. This tighter agreement could be attributed to more stable physiological conditions within this group, which might lead to more consistent readings between the two indices. The narrower limits of agreement in this group suggest less variability and more predictability in the measurement of oxygenation, making OSI a reliable surrogate for OI in clinically stable infants. In contrast, the analysis for non-surviving infants shows a higher mean difference with much wider limits of agreement. These findings suggest significant discrepancies between OI and OSI in this group. The wider limits could be due to the more severe, fluctuating clinical states seen in these infants. These differential findings underscore the need for careful interpretation of OSI in varying clinical scenarios. While OSI can be a valuable tool in stable patients, its use in critically ill infants requires a cautious approach, considering the potential for significant physiological variances that can alter its accuracy. Further research into these variations could help clarify the underlying causes of the observed discrepancies and improve the clinical application of OSI, particularly in critical care environments.

We have shown that significant correlations exist among various OIs and OSIs on the first day for a cohort of extremely preterm infants. Spearman's rho correlation coefficients confirm strong associations between mean, best (lowest), and highest values of both the OI and OSI. This indicates that changes in one index typically align with changes in the others, demonstrating that each index, whether it captures mean, peak, or optimal values, accurately reflects the respiratory status of these infants on the first day. The consistent relationships among these indices provide clinicians with reliable tools for assessing and monitoring the respiratory function of extremely preterm infants. Furthermore, we have shown that various OIs and OSIs in the first 24 h, provides significant predictive insight into mortality in the first 7 days of life. These measurements effectively distinguish survival outcomes, indicating their utility in neonatal intensive care settings.

We have also shown that infants diagnosed with BPD at 36wks PMA consistently exhibit higher values of both OI and OSI from the second day of life compared to those without BPD. The consistent difference in these indices between infants who did and did not develop BPD underscores their potential utility as early indicators for BPD development. Recognizing these elevated values early may allow clinicians to implement more targeted interventions, potentially improving the long-term respiratory outcomes. Furthermore, our analysis of the daily OI and OSI averages highlights their increasing reliability as predictors of BPD risk from the fourth day of life. While the predictive capacity of OI and OSI for BPD may seem expected, our study provides additional insight by examining these indices during the first week of life among extreme preterm infants and comparing patterns between infants who developed BPD and those who did not. Although we have not demonstrated that interventions guided by OI and OSI prevent or reduce BPD, the observed differences between groups suggest that using these indices as early markers could help clinicians identify and manage at-risk infants more effectively. In doing so, our study addresses a gap in the early prediction of BPD and lays groundwork for future research on intervention strategies informed by these measures.

The heterogeneity of our study population, which includes both preterm infants on mechanical ventilation and those on non-invasive ventilation, reflects regional differences in practices and patient populations. Unlike European cohorts, our institution has a higher number of preterm infants intubated in the delivery room, particularly those under 26 weeks of gestation, due to their immediate stabilization needs. While the Less Invasive Surfactant Administration (LISA) technique has been recently introduced for infants over 26 weeks, it is still in the early stages of implementation. As experience with LISA grows, it is expected to be more broadly applied, potentially reducing intubation rates in the future. This ongoing transition highlights our commitment to advancing neonatal care and updating clinical practices.

Recently the utility of the OSI has been highlighted in both term and preterm infants, demonstrating its effectiveness in assessing respiratory status (14, 19–21). In a cross-sectional study from Tehran (14), the diagnostic accuracy of OSI for RDS in preterm neonates was confirmed with high sensitivity and specificity, reinforcing that early, accurate OSI assessments can significantly influence clinical decision-making (14). Another retrospective study involving 68 infants revealed a strong correlation between OSI and OI, with a correlation coefficient of 0.90 and an effective predictive equation of OI = (2.3 × OSI) - 4 for OI levels under 40 (19). A similar study conducted in Vietnam with mechanically ventilated infants, both term and preterm, showed a strong correlation and high concordance between the OI and the OSI (20) Another study involving 282 moderately preterm infants revealed that early assessments of the Respiratory Severity Score (RSS) alongside OSI could effectively predict the need to escalate non-invasive respiratory support (NIRS) such as CPAP or NIMV to mechanical ventilation (21).

This study's major strength lies in its focus on a well-defined cohort of extremely preterm infants, offering detailed insights into the early respiratory status of extremely premature infants. The extensive data collection over the first seven days of life allows for a robust analysis of OI and OSI correlations and their predictive value regarding clinical outcomes. However, the study also faces limitations that must be acknowledged. Its observational design restricts the ability to draw causal inferences from the data. The sample size, while adequate for statistical power, is relatively small given the complexity and variability of neonatal conditions, which may limit the generalizability of the findings to all preterm populations. It is also important to acknowledge the inherent limitations of pulse oximetry, which may affect its accuracy and reliability under certain conditions. Pulse oximetry tends to have reduced precision at the highest oxygen saturation levels and in hypoxic states, where the correlation with arterial oxygenation may weaken. Additionally, factors such as hypothermia, acidosis, motion artifacts, and the proportion of fetal hemoglobin can further impact the accuracy of SpO₂ measurements. These factors are particularly relevant in neonatal populations, where such physiological variations are more common. Although we used the Masimo SET system and NeoPT sensors, which are designed to minimize motion artifacts and improve accuracy, these limitations remain inherent to the technology and may have influenced the precision of our data. Future studies should consider these limitations when interpreting oxygenation indices derived from pulse oximetry. Furthermore, the focus exclusively on extremely preterm infants may exclude relevant insights applicable to late preterm or term neonates. Additionally, while this study provides valuable insights into the risk factors and outcomes of BPD, it does not account for the role of hemodynamically significant patent ductus arteriosus (HsPDA), known to influence BPD outcomes. Another limitation is the study's setting in a single center, which may introduce bias related to specific clinical practices or demographic factors not representative of other regions or populations. Finally, while the study captures data from the first week of life, longer-term follow-up could provide more comprehensive insights into the predictive value of early respiratory indices on long-term outcomes. These factors should be considered when interpreting the study results and planning future research to validate and extend these findings.

## Conclusion

This study substantiates the strong correlation and predictive utility of the OI and OSI in assessing respiratory function in extremely preterm infants, affirming their value in early clinical assessments. By demonstrating consistent correlations across different clinical conditions and providing a quantitative basis for predicting survival and respiratory outcomes, our findings highlight the potential of OI and OSI as effective tools for timely intervention and management of respiratory complications in this high-risk population. While the results are promising, cautious interpretation is required, especially in critical cases, underscoring the need for further research to refine these indices for broader clinical application and to optimize neonatal care strategies.

## Data Availability

The raw data supporting the conclusions of this article will be made available by the authors, without undue reservation.
